# Knowledge and Implementation of the New European Guide in the Management of Arterial Hypertension. The Cigema Survey

**DOI:** 10.3390/ph2020011

**Published:** 2009-07-28

**Authors:** Leopoldo Pérez de Isla, Luis Miguel Ruilope, Alejandro de la Sierra, José Zamorano

**Affiliations:** Instituto Cardiovascular, Hospital Clínico San Carlos, Profesor Martín Lagos, S/N, Madrid 28040, Spain; Email: leopisla@hotmail.com (L.P.I.); lmruilope@yahoo.es (L.M.R.); adelasierra@hotmail.com (A.S.)

**Keywords:** arterial hypertension, Delphi method, guidelines

## Abstract

Knowledge of guideline implementation pitfalls allows anticipation and solving of problems and may help to promote implementation. The aims of this study were: 1) to find out how much is known among medical professionals about the recommendations for the Management of Arterial Hypertension; 2) to study in depth the extent of implementation and 3) to evaluate the manner in which this guide will be applied to daily medical practice. The Delphi method was used for this work. The total estimated sample size was 2,250 physicians. The carefully selected experts answered questionnaires in two or more rounds. The final sample size was 2,475 physicians. Results of the study are detailed in the article. Among the resultsIt is noteworthy that the guide is viewed as needed among all those who have been interviewed and this agreement about its need is generalised and that the improvement in medical practice, together with individual treatment and cardiovascular risk stratification are viewed positively in opinions reached by consensus by the majority of physicians, regardless of whether they are specialists or general practitioners. The main results of this study emphasize the fact that physicians need a guideline for the management of hypertensive patients and that most of physicians agree with them. The new guidelines on arterial hypertension management are widely known among physicians and there appears to be a global agreement regarding the need for the implementation of the new recommendations.

## Introduction

The implementation of Practice Guidelines is a very important activity of every scientific society. In order to disseminate their use, one of the most important concerns is the knowledge of its use within the target population. The knowledge of implementation pitfalls allows solving any potential problem and may help to promote implementation. 

Recently, the new European Society of Cardiology (ESC) Guidelines on Arterial Hypertension (AH) have been published. These new guidelines up-date the previous version, published in 2003, and it includes some changes in different aspects concerning the management of arterial hypertension. All these changes have been based on the new scientific evidence that has appeared during the last four years. 

This document establishes new therapeutic goals for management and treatment of arterial hypertension. Nevertheless, there are some still unknown questions: 1) are these new guidelines on arterial hypertension really known by physicians? 2) In the case where they know the new recommendations, are they really followed in daily clinical practice? 3) Are there differences in the implementation of these new guidelines between specialists and general practitioners? 4) Is there any significant difference in the implementation of the new guidelines among the different public health systems in the different areas of Spain? 5) Which are the expectations of physicians regarding the new guidelines? In order to answer these questions, the study reported herein was designed and carried-out.

The aims of this study were: 1) to find out how much is known about the recommendations outlined in the new European Guide in the Management of Arterial Hypertension among medical professionals, placing emphasis on those aspects which have been changed with respect to the previous edition; 2) to study in depth the extent of implementation thereof with a view to establishing those aspects which are being applied at the moment; 3) to evaluate the manner in which this guide will be applied to daily medical practice. 

## Results and Discussion

### Sample size estimation

Sample size estimation, according the previously mentioned criteria, was: Area 1: 363 subjects; Area 2: 378 subjects; Area 3: 376 subjects; Area 4: 376 subjects; Area 5: 377 subjects; Area 6: 380 subjects. The total estimated sample size was 2,250 subject, and the final sample size was 2,475 subjects. The main results of the study are detailed below and in the corresponding figures. In order to obtain an adequate sample size, the following aspects were considered: 1) There is a population of 37,619 people divided into six areas (population size: Area 1: 3,919 subjects; Area 2: 6,896 subjects; Area 3: 6,147 subjects; Area 4: 6,295 subjects; Area 5: 6,601 subjects; Area 6: 7,761 subjects); 28,806 of these were general practitioners and 8,813 specialists; 2) Populations were considered as “finite”; 3) The study questions aim was to determine the degree of agreement on a subject by using a discrete graduation (from 0 to 5); 4) A conservative management, considering the maximal dispersion, was used for the sample size calculation, due to the lack of previous data regarding the estimated dispersions; 5) The confidence level was 0.95; 6) The error intervals were 0.05; 7) The sample size obtained was increased 10%; 8) Specialists were weighted by 1.33 and general practitioners by 1.0.

### Block 1: Need for the guidelines

**Figure 1:**
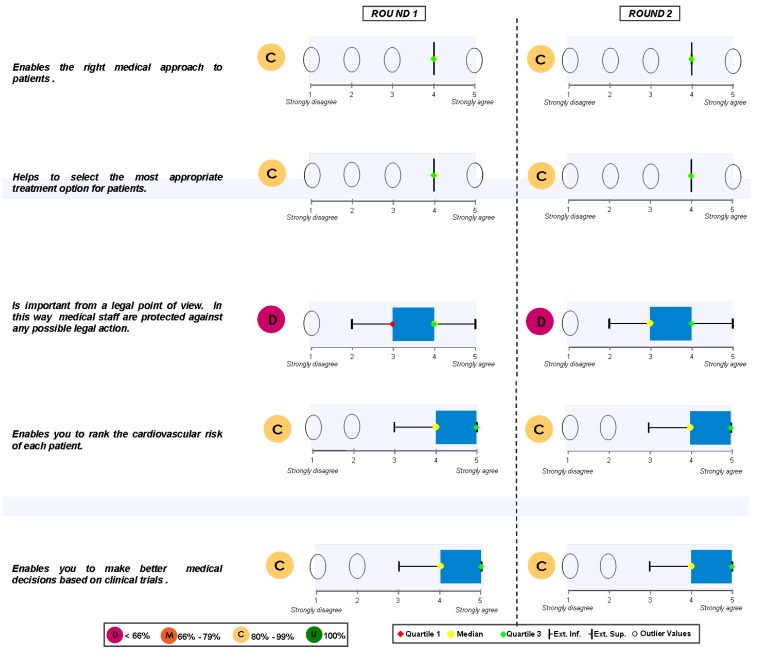
Answers to the question “from your own experience, the guide ESH/ESCH 2007 in the management of arterial hypertension…”.

**Figure 2:**
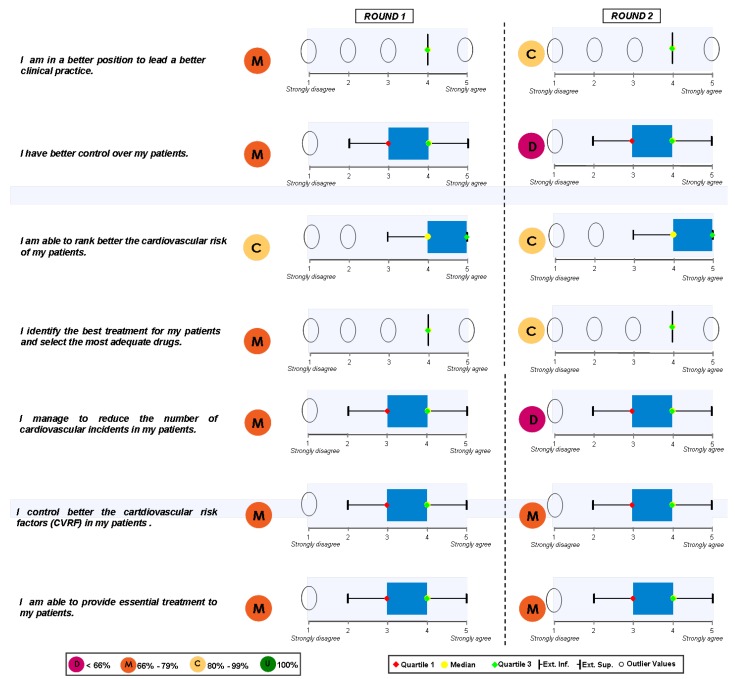
Answers to the question “in applying the guide ESH/ESC 2007 to the management of arterial hypertension in the treatment of patients…”.

The results regarding this first question are depicted in Figure 1 and Figure 2. These results show that it is very clear that the guide is needed among all those who have been interviewed and its agreement to its need is generalised. 

There were also no controversial replies in respect to an appropriate medical diagnosis as well as the selection of a therapeutic option. Nevertheless, there are some controversial replies, but positive ones, with regards to the ranking of cardiovascular risks and the decision making process based on clinical trials. And, finally, the position of those interviewed regarding the legal need for its publication is both controversial and neutral. Those interviewed had no clear opinion and did not take sides. When round 1 and round 2 were compared, no significant changes between them were found. 

### Block 2: Implementation of the Guidelines

**Figure 3:**
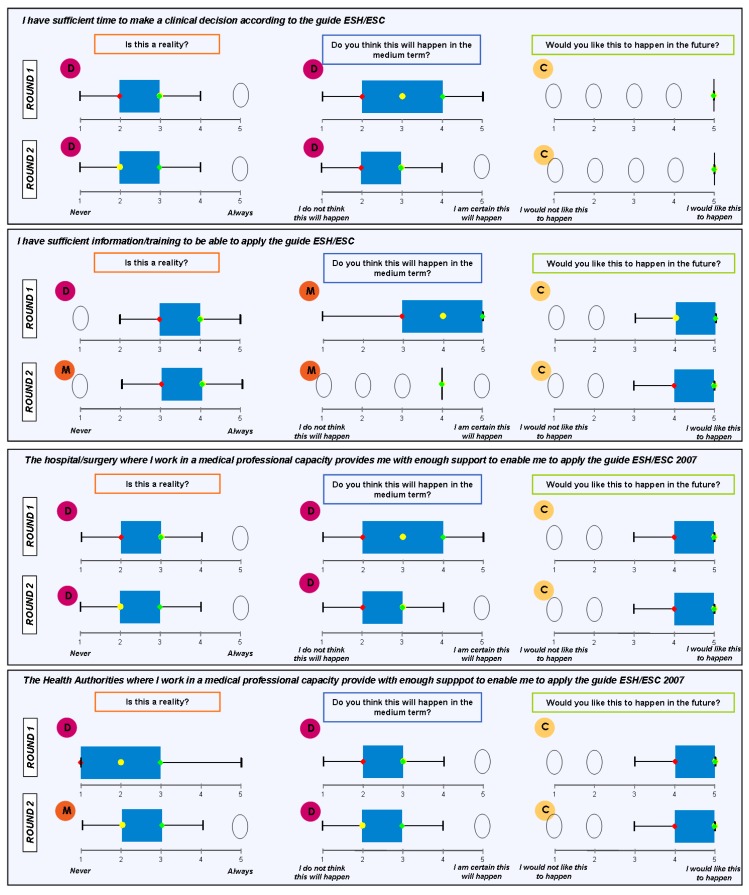
Opinions given by physicians to various statements.

**Figure 4:**
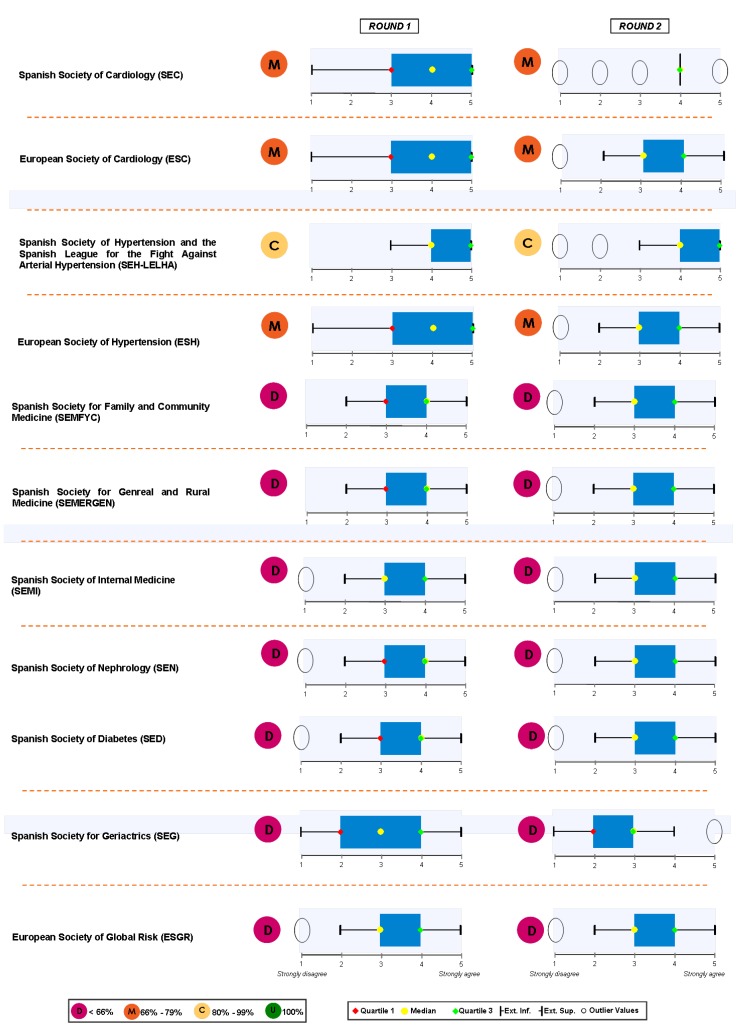
Answers to the question “which of the following scientific societies support more the dissemination and understanding of the guide ?”.

The answers for this question are shown in Figure 3 and Figure 4. The main results show that: 1) all sorts of decisions are to be found regarding the implementation of the guide. Some are generally sustained and some of them have been reached by consensus; 2) The improvement in medical practice together with individual treatment and the cardiovascular risk stratification attract positive opinions and opinions reached by consensus; 3) The improvement of cardiovascular risk frequency gets general approval and occasionally neutral-positive opinions; 4) Finally patient control and reduction in cardiovascular episodes results in discrepancy and neutral opinions. 

When round 1 and round 2 are compared, changes in every sense are found: improvement in clinical practice and individual patient care shift from widely supported opinions to opinions reached by consensus. Furthermore, improvements in patient control and reduction in cardiovascular episodes shift from widely supported opinions to controversial ones. The rest of the replies did not change.

**Figure 5:**
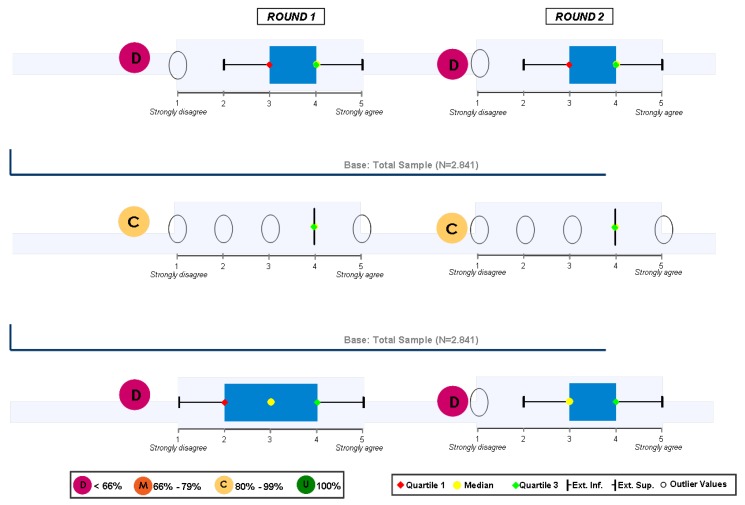
Answers to the question “would you consider the aims outlined in the guide ESH/ESC 2007 in relation to the management of arterial hypertension to be attainable?”.

### Block 3: Application of the guidelines

**Figure 6:**
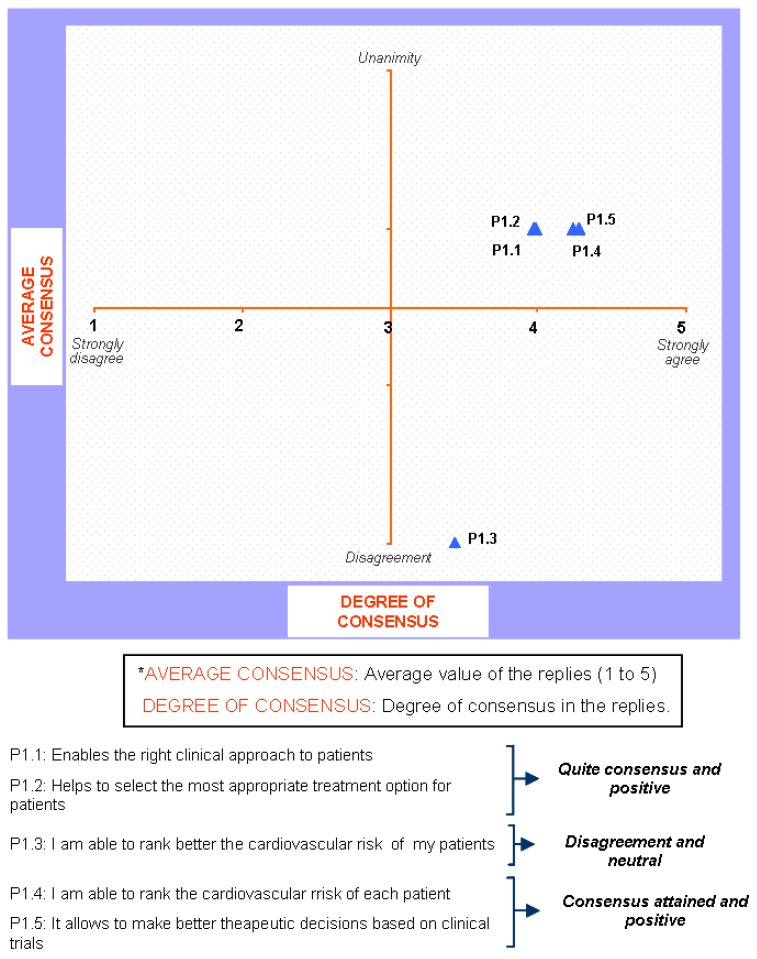
Conclusions: need for the ESH/ESC 2007 guidelines.

**Figure 7:**
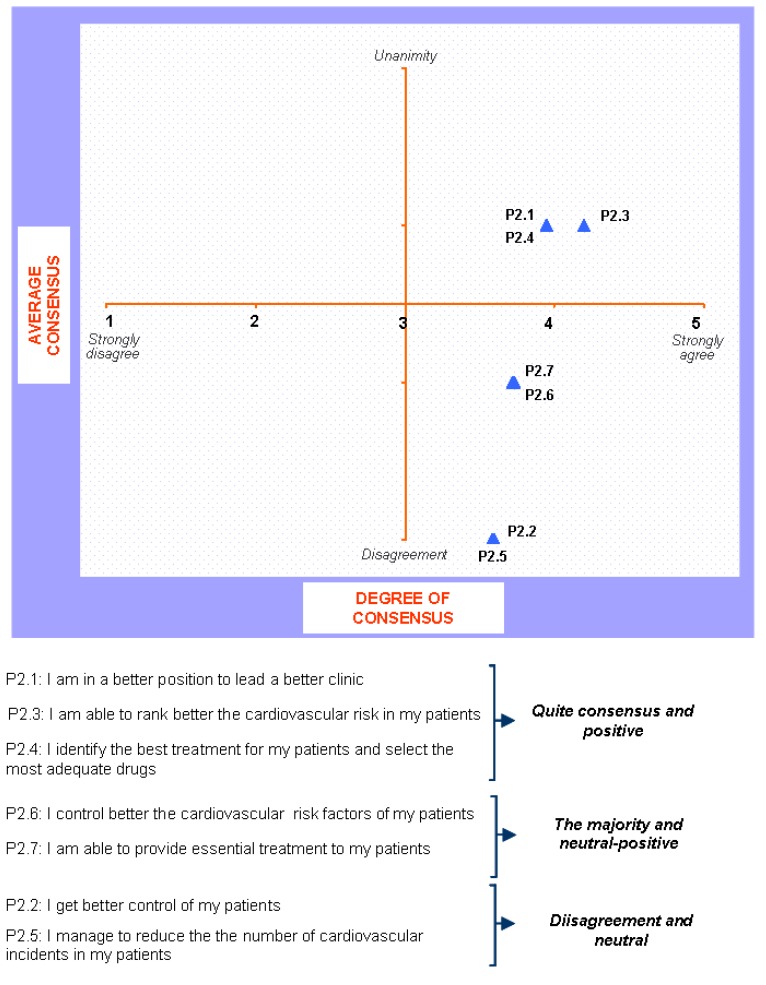
Conclusions: implementation of the ESH/ESC 2007 guidelines.

**Figure 8:**
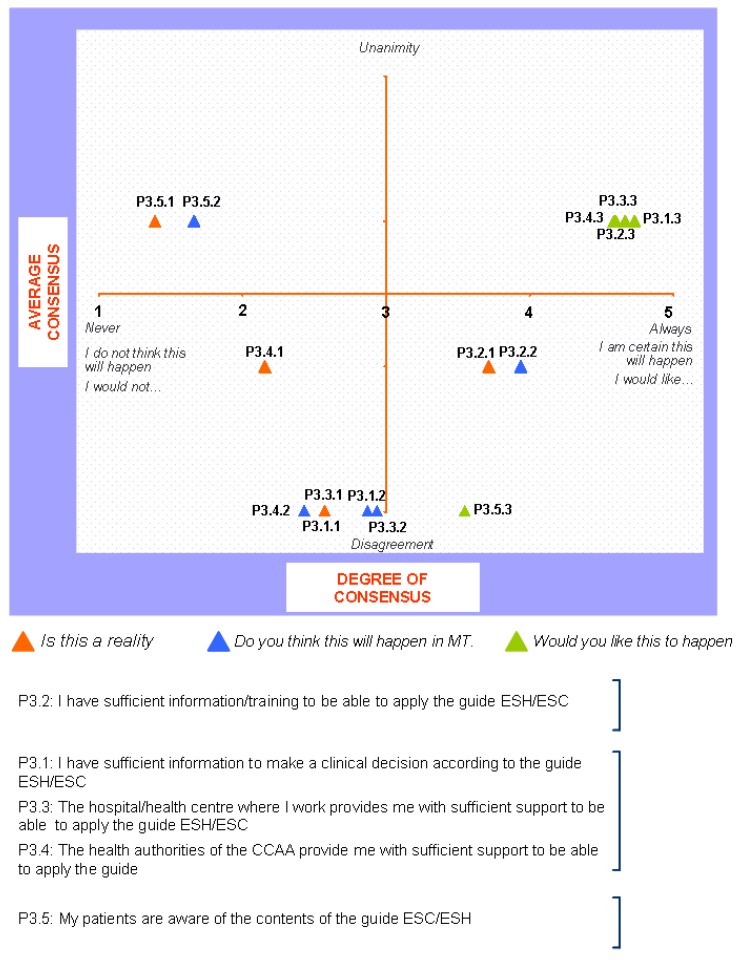
Conclusions: application of the ESH/ESC 2007 guidelines.

**Figure 9:**
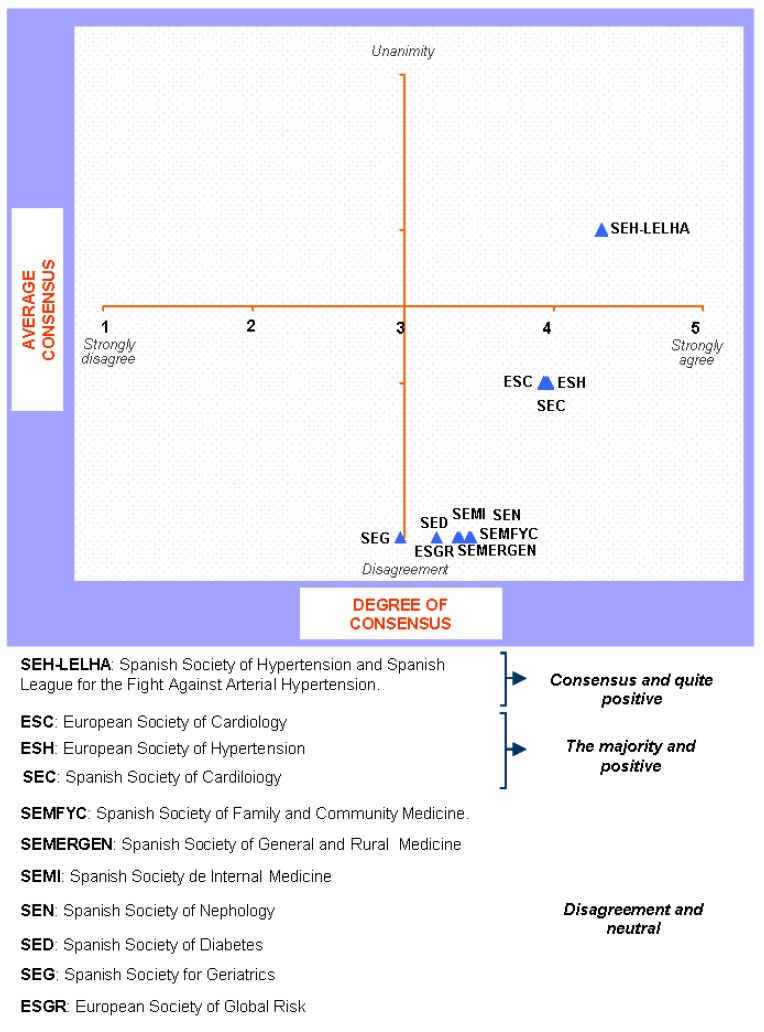
Conclusions: Scientific Societies.

**Figure 10:**
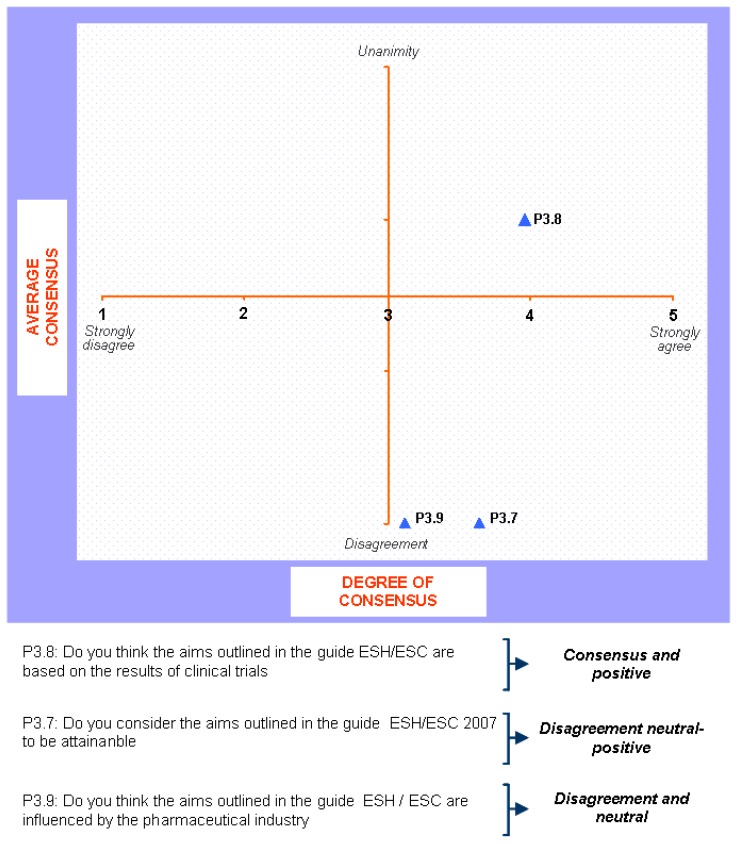
Conclusions: application of the ESH/ESC 2007 guidelines.

The findings for this point (seen in Figure 5, Figure 6, Figure 7, Figure 8, Figure 9 and Figure 10) were: 1) The potential application of the guide in relation to information from public authorities shows a somewhat agreed pattern; 2) Common grounds have been met insofar as the available information to apply the guide is concerned; 3) There are opposite or neutral positions as regards information available to make a clinical decision, as well as centre support to apply the guide. Those interviewed demanded more support from the administration; 4) there is general disagreement as far as patient’s current knowledge of the guide is concerned. The patients do not know about the guide and those interviewed have no real interest in patients knowing about it; 5) Positions were very positive in respect of the “wish issues” discussed with the exception of patient’s knowledge of the guide; 6) There is no agreement as far as dissemination and understanding of the guide among Scientific Societies is concerned, most likely due to ignorance of those interviewed and being unlinked to the remit of these bodies; 7) There appears to be three different positions: General consensus and quite positive opinion regarding the Spanish Society of Hypertension-Spanish League Against Hypertension (SEH-LELHA), majority and positive opinion regarding ESC, Spanish Society of Cardiology and European Society of Hypertension and the third and large group, that is formed by the rest of the societies which have neutral and deferring opinions; 8) As far as this part of the guide is concerned, the application of the guide is faced with both deferring positions and consensus; 9) On the one hand there is widespread agreement in relation to the clinical trials and the aims of the guide; 10) On the other hand no agreement has been reached concerning the goals and the influence of the pharmaceutical industry.

**Figure 11:**
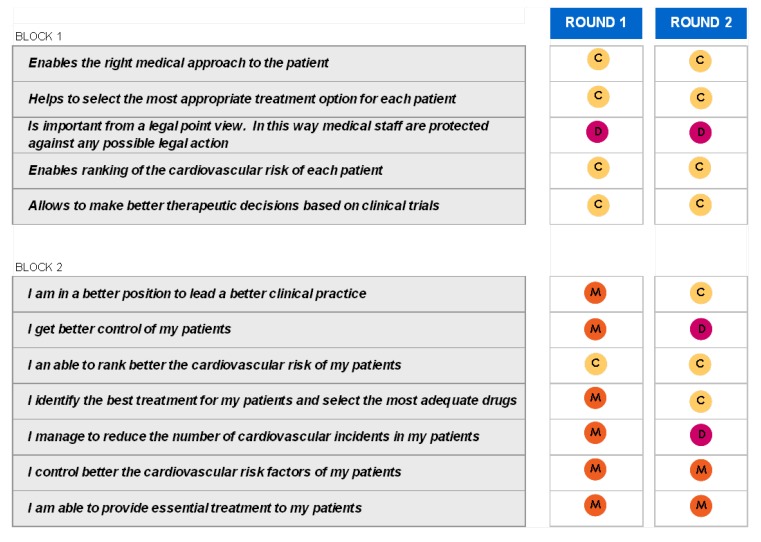
Table summary. Blocks 1 and 2.

**Figure 12:**
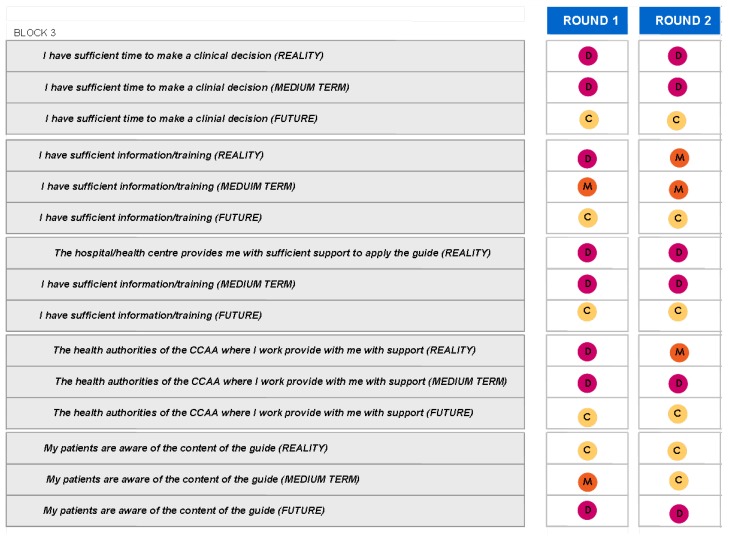
Table summary. Block 3-i.

**Figure 13:**
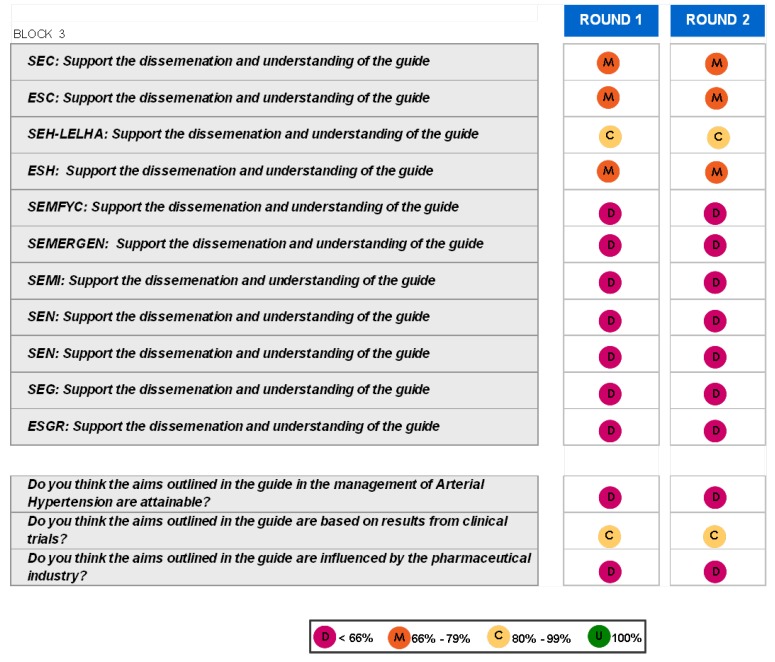
Table summary. Block 3-ii.

**Figure 14:**
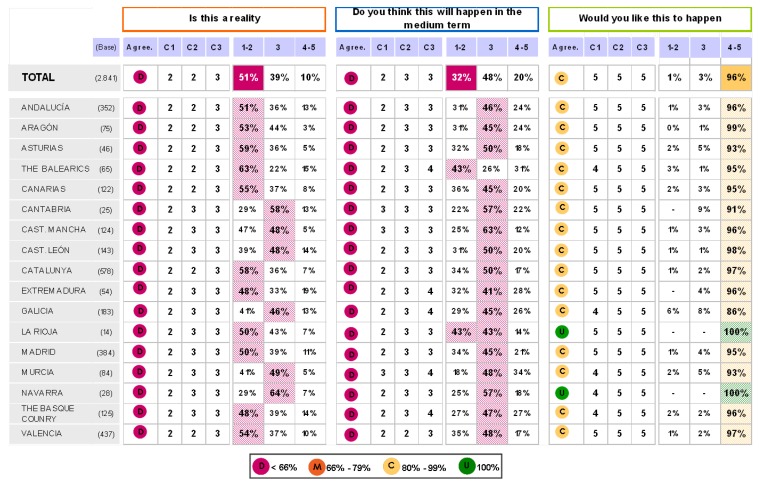
Results after segmentation by areas. Answers to the question “I have sufficient time to make medical decisions according to the ESH/ESC guidelines”.

**Figure 15:**
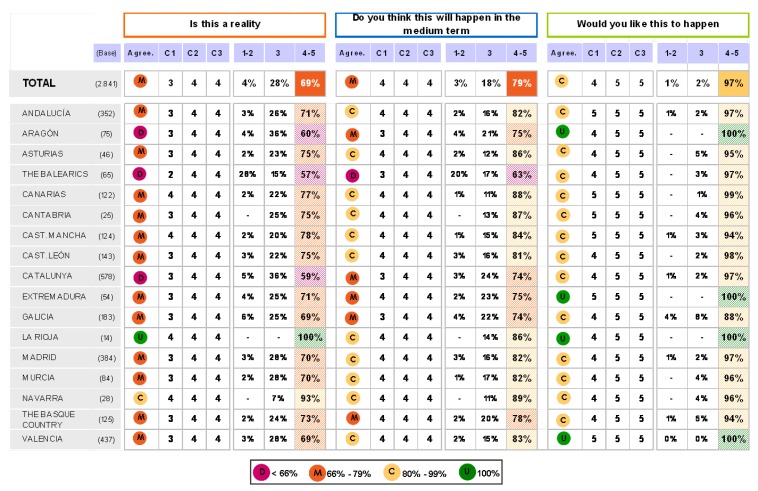
Results after segmentation by areas. Answer to the question “I have sufficient information/training to be able to apply the ESH/ESC guidelines”.

**Figure 16:**
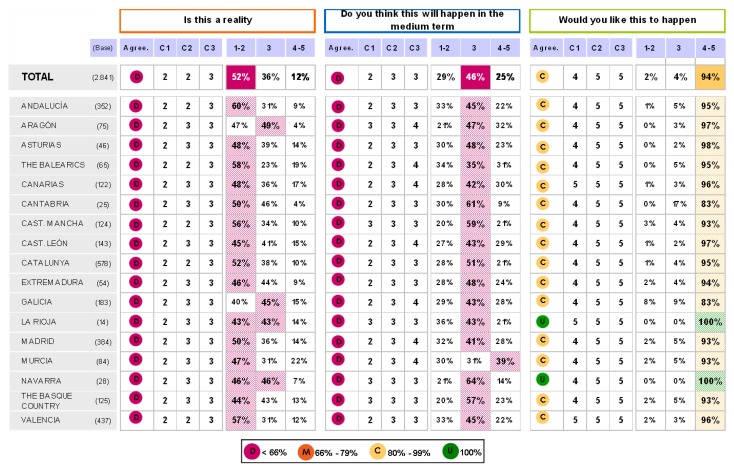
Results after segmentation by areas. Answer to the question “the hospital/health centre where I work provides me with sufficient support to be able to apply the ESH/ESC 2007 guidelines”.

**Figure 17:**
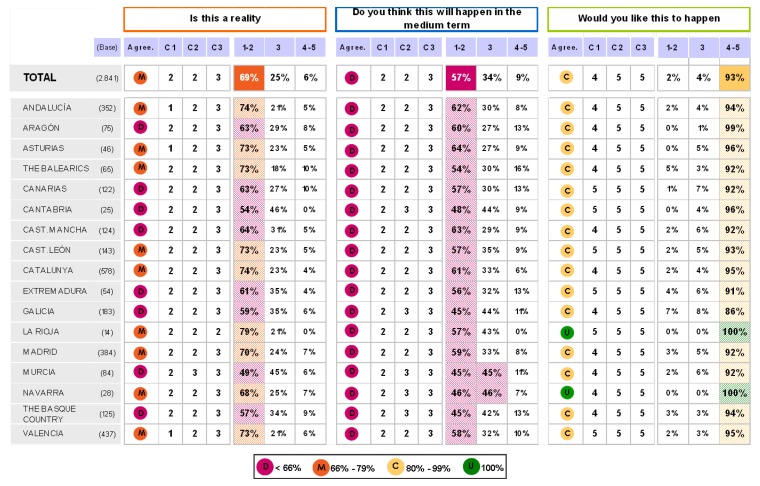
Results after segmentation by areas. Answer to the question “the health authorities of the different areas where I work provide me with sufficient support to be able to apply the ESH/ESC 2007 guidelnes”.

**Figure 18:**
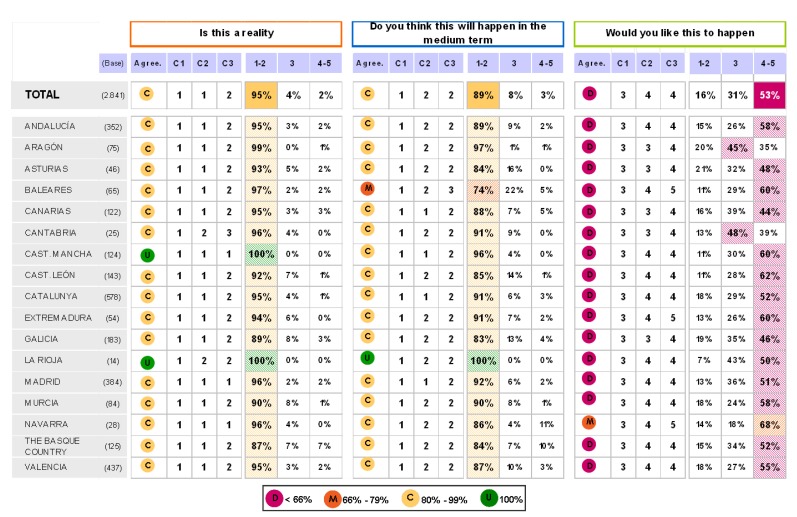
Results after segmentation by areas. Answer to the question “my patients are aware of the ESH/ESC 2007 guidelines”.

**Figure 19:**
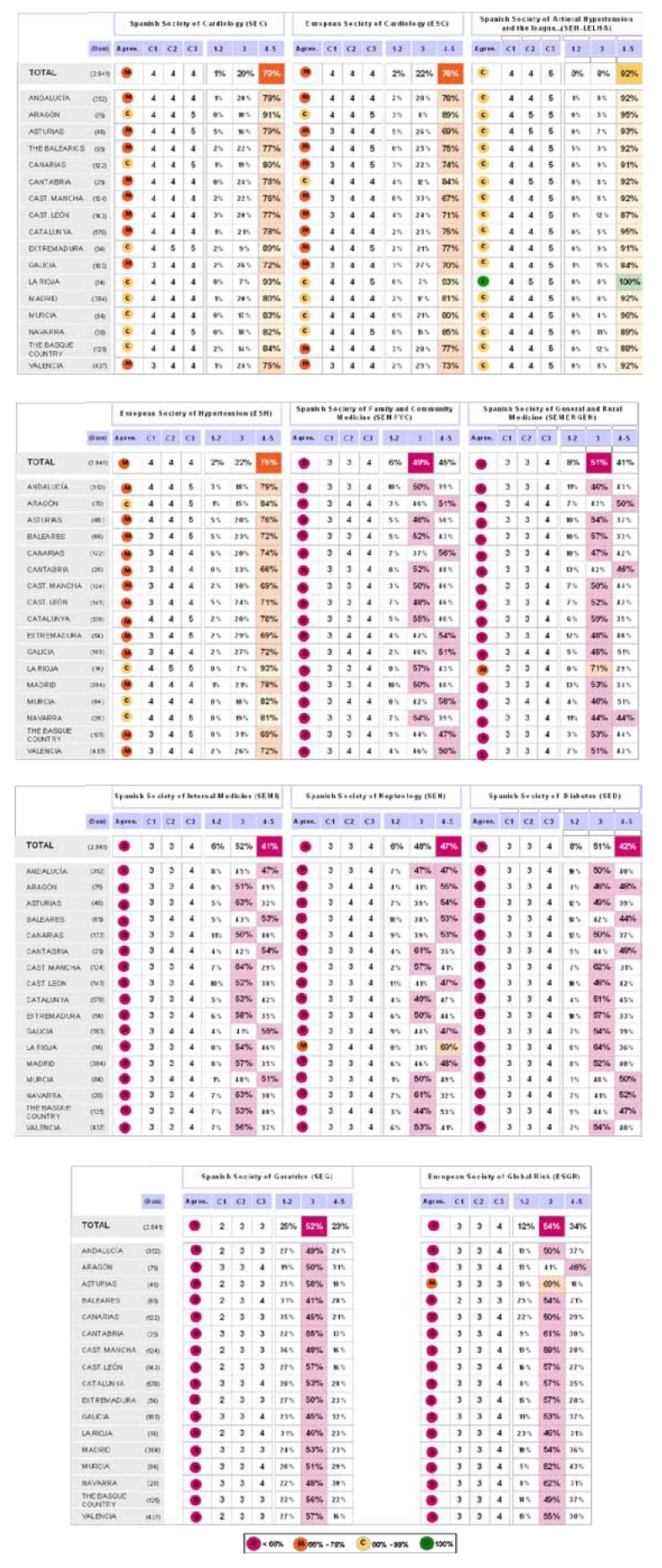
Results after segmentation by areas. Answer to the question “which of the following scientific societies support more the dissemination and understanding of the guidelines?”.

**Figure 20:**
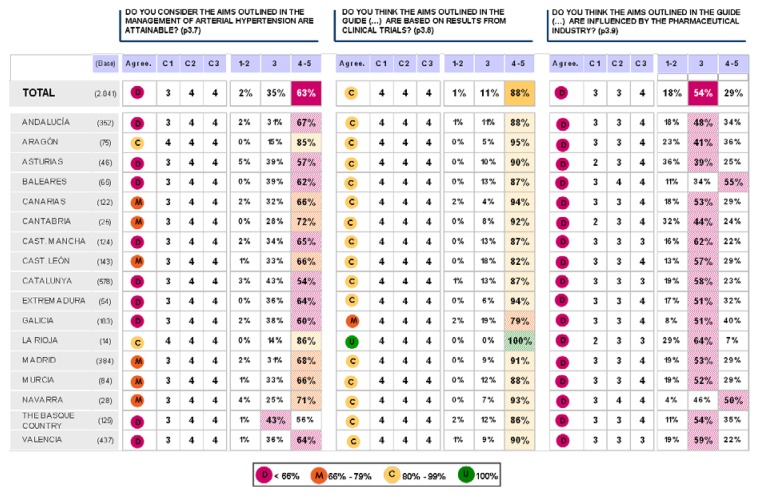
Application of the guidelines.

When round 1 and round 2 were compared, no changes were noted in respect to the first round. The differences between rounds are few, such as in the case of Scientific Societies and no changes in the degree of consensus has been noted. There were only slight modifications and there were only two differences: 1) The patient’s knowledge and 2) The fact that the authorities support for those interviewed as far as the application of the guide is concerned; 3) there is less of a spread in replies, which is hardly significant; 4) A lesser degree of disagreement has been recorded regarding the influence of the pharmaceutical industry in respect of the aims of the guide. Summarized results of the global interviewed population and results divided into the different regions are shown in Figure 9, Figure 10, Figure 11, Figure 12, Figure 13, Figure 14, Figure 15, Figure 16, Figure 17, Figure 18, Figure 19, Figure 20.

## Discussion

The new arterial hypertension guidelines try to provide the most complete and balanced recommendations for the management of this epidemic disease [1]. They update the 2003 guidelines [3], after a critical and extensive review of the new scientific data. Nevertheless, the gap between the expert’s recommendations and the real control of blood pressure in medical practice is not well known and some studies show that an important proportion of hypertensive patients are unaware of their condition or if they are aware they do not follow any treatment [4,5,6]. Furthermore, the therapeutic goal is seldom achieved, regardless the patient is followed-up by a specialist or by a general practitioner [7,8]. 

A global acceptance of the guidelines by scientific societies and physicians is a prerequisite to promote management implementation, according the recommendation of the new guidelines [1]. Furthermore, the successful implementation of the recommendations given in the guidelines requires awareness of the frontiers between recommendations and clinical practice. Thus, a deep insight into knowledge and acceptance by physicians is of great interest and it was the reason to carry out this study, which was designed in order to answer some important questions regarding the new guidelines on arterial hypertension. To our knowledge, there is no other study focused on this subject. The main aim of this study were to assess the degree of knowledge about the new recommendations, the application of theses new arterial hypertension management advice and to evaluate the manner in which these guidelines are applied to daily clinical practice. The results of the study show very interesting results. These results may help to the scientific community to design different strategies in order to increase the degree of knowledge and application of the new recommendations.

### The Delphi method

The name "Delphi" derives from the Oracle of Delphi. This method is based on the assumption that group judgments are more valid than individual judgments. The main characteristics of the Delphi method are:

1) Structuring of information flow: The initial contributions from the experts are collected in the form of answers to questionnaires and their comments to these answers. The panel director controls the interactions among the participants by processing the information and filtering out irrelevant content. This avoids the negative effects of face-to-face panel discussions and solves the usual problems of group dynamics.
2) Regular feedback: Participants comment on their own forecasts, the responses of others and on the progress of the panel as a whole. At any moment they can revise their earlier statements. While in regular group meetings participants tend to stick to previously stated opinions and often conform too much to group leader, the Delphi method prevents it.
3) Anonymity of the participants: Usually all participants maintain anonymity. Their identity is not revealed even after the completion of the final report. This stops them from dominating others in the process using their authority or personality, frees them to some extent from their personal biases, allows them to express their opinions in a free manner and encourages critique and admitting errors.

The person in charge of the coordination of the Delphi method is known as a facilitator, and facilitates the responses of their panel of experts, who are selected for a reason, usually that they hold knowledge on an opinion or view. The facilitator sends out questionnaires, surveys etc. and if the panel of experts accept, they follow instructions and present their views. Responses are collected and analyzed and then common and conflicting viewpoints are identified. If consensus is not reached, the process continues through thesis and antithesis, to gradually work towards synthesis, and building consensus.

### Study results

Among the different results of the present study, the following aspects are noteworthy: it is very clear that the guide is needed among all physicians, regardless of whether they are specialists or general practitioners; the agreement to the need for the guidelines is generalised; the potential application of the guide in relation to information from public authorities showed an agreed pattern; more support from the administration in needed in order to apply the new guidelines; There is no agreement as far as dissemination and understanding of the guide among Scientific Societies is concerned; and no agreement has been reached concerning the goals and the influence of the pharmaceutical industry.

There have been some cases when the method produced poor results. It should be taken into account that there are some areas such as science in which the degree of uncertainty is so great that exact and always correct predictions are impossible, so a high degree of error is to be expected. Another particular weakness of the Delphi method is that future developments are not always predicted correctly by consensus of experts. Despite these limitations, the Delphi method is nowadays a widely accepted forecasting tool and has been used successfully for studies in different areas.

## Experimental Section

### The Delphi method

This method is based on the assumption that group judgments are more valid than individual judgments. The Delphi method is a systematic, interactive forecasting method which relies on a panel of independent experts. The carefully selected experts answer questionnaires in two or more rounds. After each round, a facilitator provides an anonymous summary of the experts’ forecasts from the previous round as well as the reasons they provided for their judgments. Thus, participants are encouraged to revise their earlier answers in light of the replies of other members of the group. It is believed that during this process the range of the answers will decrease and the group will converge towards the "correct" answer. Finally, the process is stopped after a pre-defined stop criteria and the mean or median scores of the final rounds determine the results. The technique can be adapted for use in face-to-face meetings.

### Study population

**Figure 21:**
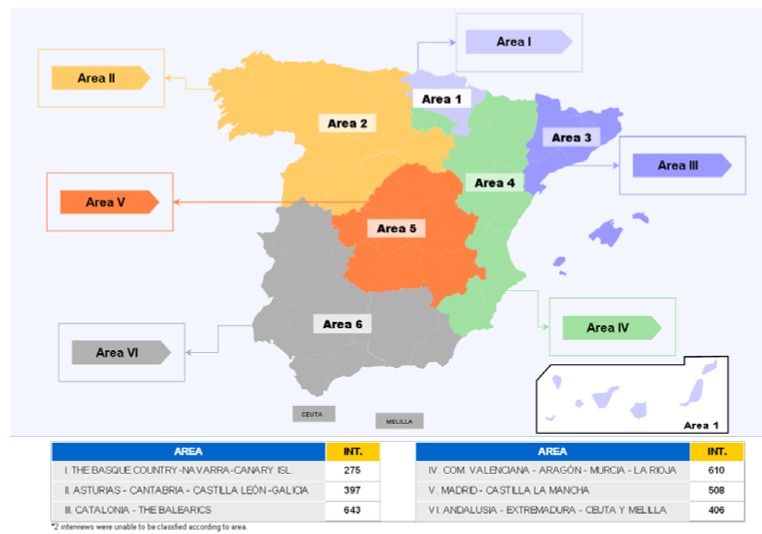
Territorial division of the interviews.

**Figure 22:**
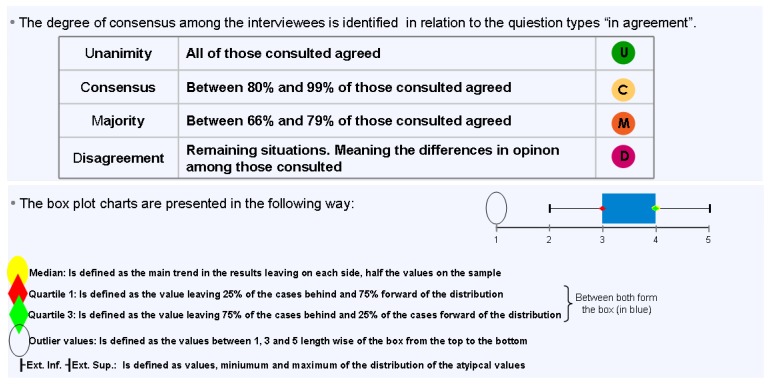
Methodology.

The scope of this study was the Spanish population. A total of 2.841 interviews were performed, according the “FACE to FACE” method. The degree of consensus among the interviews was identified in relation to the question types “in agreement”. The box plot charts were constructed according the way described in Figure 21. The level of the distribution of the interviews among the different geographic areas in Spain is detailed in Figure 22. 

## Conclusions

Although there are still some limitations regarding the knowledge and implementation of the new guidelines on arterial hypertension, the main results of this study emphasize the fact that physicians need a guideline for the management of hypertensive patients and that most of physicians agree with them. The new guidelines on arterial hypertension management are widely known among physicians and there is a global agreement regarding the need of the implementation of the new recommendations.
